# Supplementary material for: Meta-Analysis of the Effect of Ventilation on Intellectual Productivity
Source: Int J Environ Res Public Health. 2023 Apr 19;20(8):5576. doi: 10.3390/ijerph20085576 (PMC10139101; doi:10.3390/ijerph20085576)
Supplement: Supplementary file 1 [file ijerph-20-05576-s001.zip › Supplementary_Methods_v2.pdf]

Supplementary Methods

Details of search strategy

PubMed strategy:

(“ventilation”[All Fields] OR “air supply”[All Fields] OR “supply air”[All Fields] OR “open air”[All Fields]) AND ((“school”[All Fields] OR “classroom”[All Fields] OR “pupil”[All Fields] OR “student”[All Fields]) OR (“office”[All Fields] OR “worker”[All Fields])) AND (“cognitive skill”[All Fields] OR “intellectual skill”[All Fields] OR “achievement”[All Fields] OR “performance”[All Fields] OR “productivity”[All Fields])

Web of Science strategy:

(“ventilation” OR “air supply” OR “supply air” OR “open air”) AND ((“school” OR “classroom” OR “pupil” OR “student”) OR (“office” OR “worker”)) AND (“cognitive skill” OR “intellectual skill” OR “achievement” OR “performance” OR “productivity”)

Scopus strategy:

TITLE-ABS-KEY (((ventilation) OR (air AND supply) OR (supply AND air) OR (open AND air)) AND (((school) OR (classroom) OR (pupil) OR (student)) OR ((office) OR (worker))) AND ((cognitive AND skill) OR (intellectual AND skill) OR (achievement) OR (performance) OR (productivity)))

Calculation of dose-response

First, we calculate the fractional change (λ) for each performance. The fractional change(λ) in this study represents the change in performance when the ventilation rate changes by 1 L/(s·person). The fractional change (λ) is calculated using (1) because the results reported in the adopted study may be based on various standards.

$$\lambda = \frac{P}{(VH - VL)} \qquad (1)$$

where P is the difference between the standardized means of the results of each study, VL is the lower ventilation rate at the performance measurement, and VH is the higher ventilation rate.

To ensure that the assumption of linearity is maintained, the midrange fractional change λ ($\lambda\, mid$), which is the change of performance at the midpoint of the ventilation rates, is calculated using (2) according to Seppänen et al. (2006).

$$\lambda\, mid = \frac{\lambda}{1 + 0.5\lambda(VH - VL)} \qquad (2)$$

Finally, $\lambda\, mid$ of each result weighted by each study's sample size is regressed on the midpoint of the ventilation rates that $\lambda\, mid$ was used to calculate.

Calculation of error rate and standard deviation (SD)

Because Coley (2012) and Peterson (2012) reported only the number of incorrect and correct answers and their respective SDs, we calculated the rate of errors and the speed of performing tasks as follows. If we take

$$x = number\ of\ wrong\ answers$$
$$y = number\ of\ correct\ answers$$

The error rate $q(x, y)$ can be calculated as follows:

$$q(x, y) = \frac{x}{(x+y)\times 100} \quad (3)$$

Here, we have

$$\frac{dq}{dx} = \frac{y}{(x+y)^2} \times 100$$
$$\frac{dq}{dy} = \frac{-x}{(x+y)^2} \times 100$$

Therefore, using the propagation of error rule (Taylor, 2000), the following holds for SD in (3).

$$S.D.of\ q(x, y) \le |\frac{100y}{(x+y)^2}| \times S.D.of\ x + |\frac{-100x}{(x+y)^2}| \times S.D.of\ y$$

From the above equation, let $S.D.of\ q(x, y)$ be the value on the right-hand side to obtain the most conservative estimate of the effect size. The total number of answers was used as an indicator of the speed of answering.

$$The\ total\ number\ of\ answers = the\ number\ of\ wrong\ answers + the\ number\ of\ correct\ answers$$
$$S.D.of\ the\ total\ number\ of\ answers = S.D.of\ the\ number\ of\ wrong\ answers + S.D.of\ the\ number\ of\ correct\ answers$$

Furthermore, as the outcome of the d2 test in Hviid (2020) was described in TOT CORR and CONC, we defined the error rate and the speed of answering and their respective SDs as follows.

$$Error\ rate = \left(\frac{1 - CONC}{N}\right) \times 100$$

$$S.D.of\ error\ rate = \left(\frac{S.D.of\ CONC}{N}\right) \times 100$$

$$The\ speed\ of\ answering\ = TOT\ CORR$$

$$S.D.of\ the\ speed\ of\ answering\ = S.D.of\ TOT\ CORR$$

In addition, the results of experiments with the same ventilation rates were integrated based on the following equation to evaluate the impact of only the change in ventilation rate in some references, which used a 2×2 crossover design where room temperature and the old and new ventilation filters were manipulated.

$$Integrated\ mean\ = \frac{res_1 \times num_1 + res_2 \times num_2}{num_1 + num_2}$$

$$S.D.of\ integrated\ result$$

$$= \sqrt{\frac{(num_1 - 1) \times {sd_1}^2 + (num_2 - 1) \times {sd_2}^2 + num_1 \times (Im - res_1)^2 + num_2 \times (Im - res_2)^2}{num_1 + num_2 - 1}}$$

The experimental results of the same test under different indoor conditions in the same literature were integrated based on the following equation.

$$Integrated\ mean\ = \frac{res_1 \times num_1 + res_2 \times num_2 + res_3 \times num_3}{num_1 + num_2 + num_3}$$

$$S.D.of\ integrated\ result$$

$$= \sqrt{\frac{(num_1 - 1) \times {sd_1}^2 + (num_2 - 1) \times {sd_2}^2 + (num_3 - 1) \times {sd_3}^2 + num_1 \times (Im - res_1)^2 + num_2 \times (Im - res_2)^2 + num_3 \times (Im - res_3)^2}{num_1 + num_2 + num_3 - 1}}$$

where num_1 is the number of experiment 1, num_2 is the number of experiment 2, num_3 is the number of experiment 3, res_1 is the result of experiment 1, res_2 is the result of experiment 2, res_3 is the result of experiment 3, sd_1 is the SD of experiment 1, sd_2 is the SD of experiment 2, sd_3 is the SD of experiment 3, and Im is the integrated mean.

Conversion of standardized mean difference (SMD) results obtained from the meta-analysis into units of the outcome measures

First, the weighted averages of the control and intervention standard deviations (SDs) from each study were obtained. The weighted SDs for each study were calculated as follows:

Weighted SD = $\frac{SD_L \times n_L + SD_H \times n_H}{n_L + n_H}$,

where SD_L is the SD of the control group, SD_H is the SD of the intervention group, n_L is the number of participants in the control group, and n_H is the number of participants in the intervention group.

Second, the study that contained the median of the weighted SDs was adopted as the outcome measures in the conversion. The median is denoted as *medSD*. If the *medSD* study did not exist because there were an even number of the studies, the study with the largest weighted SD among those lower than *medSD* was adopted.

Finally, the restatement of outcomes (RES) is calculated using the following formula.

RES = $\frac{SMD \times medSD}{medOutcome_L} \times 100$

where SMD is the SMD result obtained in the meta-analysis, and $medOutcome_L$ is the outcome of the control group in the adopted study.
