# Supplementary material for: Meta-Analysis of the Effect of Ventilation on Intellectual Productivity
Source: Int J Environ Res Public Health. 2023 Apr 19;20(8):5576. doi: 10.3390/ijerph20085576 (PMC10139101; doi:10.3390/ijerph20085576)
Supplement: Supplementary file 1 [file ijerph-20-05576-s001.zip › Supplimental_table_S2_S3.pdf]

Supplementary Table S2. Reasons for Risk of Bias Rating.

Lead author	design	Exposure assessment	Outcome assessment	Confounding	Completeness of outcome data	Selective outcome reporting	Conflict of interest	Other
Wargocki, P. (2007a)[28]	Case-crossover	Single quality controlled monitor, assumed <25% missing	The test was accurately scored based on objective criteria	Adjusting the results for these effects of increased familiarity with the exercise, fatigue, or differences between test versions	Missing data not expected to introduce substantial bias	All pre-specified outcomes outlined in the methods reported completely in a pre-specified way and no outcomes reported that were not pre-specified	Indirect evidence suggests study was free of conflict	None
Wargoki, P. (2007b)[29]	Case-crossover	Single quality controlled monitor, assumed <25% missing	The test was accurately scored based on objective criteria	Adjusting the results for these effects of increased familiarity with the exercise, fatigue, or differences between test versions	Missing data not expected to introduce substantial bias	All pre-specified outcomes outlined in the methods reported completely in a pre-specified way and no outcomes reported that were not pre-specified	Indirect evidence suggests study was free of conflict	None
Coley, D. (2007)[30]	Case-crossover	Single quality controlled monitor, assumed <25% missing	The test was accurately scored based on objective criteria	Lacking the effort to minimize confounding, e.g., the effect of increased familiarity with the exercise	Missing data not expected to introduce substantial bias	All pre-specified outcomes outlined in the methods reported completely in a pre-specified way and no outcomes reported that were not pre-specified	Indirect evidence suggests study was free of conflict	None
Petersen. S. (2016)[31]	Case-crossover	Single quality controlled monitor, assumed <25% missing	The test was accurately scored based on objective criteria	To minimize the effect of increased familiarity with the exercise, a rehearsal period was added prior to the baseline and crossover experiment	Missing data not expected to introduce substantial bias	All pre-specified outcomes outlined in the methods reported completely in a pre-specified way and no outcomes reported that were not pre-specified	Indirect evidence suggests study was free of conflict	None
Hviid, C. (2020)[32]	Case-crossover	Single quality controlled monitor, assumed <25% missing	The test was accurately scored based on objective criteria	Taking special precautions to minimize the effect of the external factors, such as solar radiation and positive learning curve, etc.	Missing data not expected to introduce substantial bias	All pre-specified outcomes outlined in the methods reported completely in a pre-specified way and no outcomes reported that were not pre-specified	Study authors make a claim denying conflicts of interest	None

Supplementary Table S3. The tasks and the type of each task in the studies included in the meta-analysis.

Lead author	Task	Type of task	Description	Outcome
Wargocki, P. (2007a)[28]	subtraction	arithmetic	the pupils subtracted two four-digit numbers.	Attempted units per min
				Percentage errors
	multiplication	arithmetic	the pupils multiplied two-digit numbers by three-digit numbers.	Attempted units per min
				Percentage errors
	addition	arithmetic	the pupils added two four-digit numbers.	Attempted units per min
				Percentage errors
	logical thinking	verbal comprehension	the pupils categorized statements describing the order of the letter pairs AB and BA as True or False.	Attempted units per min
				Percentage errors
	acoustic proof reading	verbal comprehension	the pupils read a text, marking the inserted errors, while listening to a recorded voice reading it aloud (10 errors were inserted per page of an otherwise correct transcript in such a way that they could not be found without listening and reading simultaneously).	Attempted units per min
				Percentage errors
	reading and comprehension	verbal comprehension	the pupils read text with choice points inserted (to determine whether the children understood the text, they had to mark one of three different words at each choice-point; all three words were correct in the immediate context of the phrase into which they had been inserted, but only one was correct in the context of the whole text).	Attempted units per min
				Percentage errors
	proofreading	verbal comprehension	the pupils read a prepared text in which four different kinds of errors had been inserted: spelling errors, two kinds of grammatical errors (one obvious in the context of the phrase in which it occurs and one correct in this context but incorrect in the wider context of the preceding text), and logical errors.	Attempted units per min
				Percentage errors
	number comparison	cognitive ability	the pupils checked columns of two seven-digit numbers against each other, the numbers being made similar or different by rotating three of the digits in the middle.	Attempted units per min
				Percentage errors
Wargoki, P. (2007b)[29]	subtraction	arithmetic	the pupils subtracted two four-digit numbers.	Attempted units per min
				Percentage errors
	multiplication	arithmetic	the pupils multiplied two-digit numbers by three-digit numbers.	Attempted units per min
				Percentage errors
	addition	arithmetic	the pupils added two four-digit numbers.	Attempted units per min
				Percentage errors
	logical thinking	verbal comprehension	the pupils categorized statements describing the order of the letter pairs AB and BA as True or False.	Attempted units per min
				Percentage errors
	acoustic proof reading	verbal comprehension	the pupils read a text, marking the inserted errors, while listening to a recorded voice reading it aloud (10 errors were inserted per page of an otherwise correct transcript in such a way that they could not be found without listening and reading simultaneously).	Attempted units per min
				Percentage errors
	reading and comprehension	verbal comprehension	the pupils read text with choice points inserted (to determine whether the children understood the text, they had to mark one of three different words at each choice-point; all three words were correct in the immediate context of the phrase into which they had been inserted, but only one was correct in the context of the whole text).	Attempted units per min
				Percentage errors
	number comparison	cognitive ability	the pupils checked columns of two seven-digit numbers against each other, the numbers being made similar or different by rotating three of the digits in the middle.	Attempted units per min
				Percentage errors
	simple reaction time	cognitive ability		Reaction time

Coley, D. (2007)[30]			The participant was instructed to press the 'YES' response button as quickly as possible every time the word 'YES' was presented on the screen. Thirty stimuli were presented with a varying inter-stimulus interval.	Accuracy
	choice reaction time	cognitive ability	Either the word 'NO' or the word 'YES' was presented on the screen and the participant was instructed to press the corresponding button as quickly as possible. There were 30 trials for which each stimulus word was chosen randomly with equal probability, with a varying inter-stimulus interval. Either the word 'NO' or the word 'YES' was presented on the screen and the participant was instructed to press the corresponding button as quickly as possible. There were 30 trials for which each stimulus word was chosen randomly with equal probability, with a varying inter-stimulus interval.	Reaction time<br>Accuracy
	digit vigilance	cognitive ability	A target digit was randomly selected and constantly displayed to the right of the screen. A series of digits was then presented in the center of the screen at the rate of 150 per min. The participant was required to press the 'YES' button as quickly as possible every time the digit in the series matched the target digit. There were 45 targets in the series and the task lasted for 3 min.	Reaction time<br>Accuracy
	picture presentation	cognitive ability	A series of 20 pictures was presented on the screen at the rate of 1 every 3 s for the participant to remember.	Reaction time<br>Accuracy
	picture recognition	cognitive ability	The original pictures plus 20 distracter pictures were presented one at a time in a randomized order. For each picture the participant had to indicate whether or not they recognized it as being from the original series by pressing the 'YES' or 'NO' button as appropriate, as quickly as possible.	Reaction time<br>Accuracy
Petersen. S. (2016)[31]	addition	arithmetic	The pupils added two four-digit numbers.	Correct answers<br>Errors
	number comparison	cognitive ability	The pupils checked columns of two seven-digit numbers against each other, the numbers being made similar or different by rotating three of the digits in the middle.	Correct answers<br>Errors
	logical thinking	verbal comprehension	The pupils categorized statements describing the order of the letter pairs AB and BA as True or False.	Correct answers<br>Errors
	reading and comprehension	verbal comprehension	The pupils read text with choice points inserted (to determine whether the children understood the text, they had to mark one of three different words at each choice-point; all three words were correct in the immediate context of the phrase into which they had been inserted, but only one was correct in the context of the whole text).	Correct answers<br>Errors
Hviid, C. (2020)[32]	d2-test	cognitive ability	The test consists of 14 lines each containing 57 symbols. Each symbol is either the letter d or p with either one or two dots (‘ or ‘‘) above or below the letter. The respondents mark only the symbol d with two dots (targets) while avoiding the other symbols (distracters).	TOT CORR<br>CONC
	baddeley test	verbal comprehension	the test combines a statement with a geometric figure and requires the pupils to categorize the statement as True or False.	Correct answers<br>Errors
	math	arithmetic	The pupils were asked to solve equations of multiplication and subtraction. The multiplication calculations were made as numbers between 2 and 5 multiplied by numbers between 10 and 50. The subtraction problems were made as numbers between 10 and 40 subtracted from numbers between 50 and 80.	Correct answers<br>Errors
